# Systematic pain assessment in nursing homes: a cluster-randomized trial using mixed-methods approach

**DOI:** 10.1186/s12877-017-0454-z

**Published:** 2017-02-28

**Authors:** Anna-Greta Mamhidir, Britt-Marie Sjölund, Birgitta Fläckman, Anders Wimo, Anders Sköldunger, Maria Engström

**Affiliations:** 1 Department of Health and Caring Sciences, Faculty of Health and Occupational Studies, University of Gävle, Gävle, Sweden; 2 Department of Public Health and Caring Sciences, Uppsala University, Uppsala, Sweden; 3 Department of Neurobiology, Care Sciences and Society (NVS), Aging Research Center (ARC), Karolinska Institutet and Stockholm University, Stockholm, Sweden; 4 Department of Health Care Sciences of Ersta, Sköndal University College, Stockholm, Sweden; 5 Division of Neurogeriatrics, Department of Neurobiology, Care Sciences and Society (NVS), Karolinska Institutet, Stockholm, Sweden; 6 Nursing Department, Medicine and Health College, Lishui University, Lishui, China

**Keywords:** Pain assessment, Pain intervention, Nursing homes, Cluster- randomized trial, Mixed-methods

## Abstract

**Background:**

Chronic pain affects nursing home residents’ daily life. Pain assessment is central to adequate pain management. The overall aim was to investigate effects of a pain management intervention on nursing homes residents and to describe staffs’ experiences of the intervention.

**Methods:**

A cluster-randomized trial and a mixed-methods approach. Randomized nursing home assignment to intervention or comparison group. The intervention group after theoretical and practical training sessions, performed systematic pain assessments using predominately observational scales with external and internal facilitators supporting the implementation. No measures were taken in the comparison group; pain management continued as before, but after the study corresponding training was provided. Resident data were collected baseline and at two follow-ups using validated scales and record reviews. Nurse group interviews were carried out twice. Primary outcome measures were wellbeing and proxy-measured pain. Secondary outcome measures were ADL-dependency and pain documentation.

**Results:**

Using both non-parametric statistics on residential level and generalized estimating equation (GEE) models to take clustering effects into account, the results revealed non-significant interaction effects for the primary outcome measures, while for ADL-dependency using Katz-ADL there was a significant interaction effect. Comparison group (*n* = 66 residents) Katz-ADL values showed increased dependency over time, while the intervention group demonstrated no significant change over time (*n* = 98). In the intervention group, 13/44 residents showed decreased pain scores over the period, 14/44 had no pain score changes ≥ 30% in either direction measured with Doloplus-2. Furthermore, 17/44 residents showed increased pain scores ≥ 30% over time, indicating pain/risk for pain; 8 identified at the first assessment and 9 were new, i.e. developed pain over time. No significant changes in the use of drugs was found in any of the groups. Nursing pain related documentation was sparse. In general, nurses from the outset were positive regarding pain assessments. Persisting positive attitudes seemed strengthened by continued assessment experiences and perceptions of improved pain management.

**Conclusion:**

The implementation of a systematic work approach to pain issues in nursing homes indicates that an increased awareness, collaboration across and shared understanding among the team members of the pain assessment results can improve pain management and lead to decreased physical deterioration or the maintenance of physical and functional abilities among NH residents. However, pain (proxy-measured) and wellbeing level did not reveal any interaction effects between the groups over time.

**Trial registration:**

The study was registered in ISRCTN71142240 in September 2012, retrospectively registered.

## Background

Pain management among residents in nursing homes (NHs) is essential for good quality care [1] and pain assessment is emphasized to be central. Despite guidelines the prevalence of chronic pain among NH residents is high and is reported to range between 33 and 83% [2–5]. Chronic pain can decrease residents’ mobility and increase dependency in activities of daily living (ADL) [6]. According to a recent study [7] the prevalence of pain was higher among residents with cognitive impairment and among residents with a greater degree of ADL-dependency. An important concern is that NH residents’ pain is often unidentified or undermanaged, which not only negatively impacts their ADLs and cognitive impairment, but also their quality of life [2, 4, 6, 8].

When making assessments of NH residents with pain or at risk for pain, it is important to include all residents, even those with cognitive impairment. Unidimensional pain self-report scales [9] and observational assessment scales [10–12] are available, with the latter scale recognizing indicators of behavioural pain among persons with communication difficulties. Barriers for improved pain management among cognitively impaired persons are for example related to inadequate staff training and infrequent use of appropriate pain assessment scales [13]. According to registered nurses (RNs), identifying pain and indicators of pain is challenging, thereby leaving residents with cognitive impairment at higher risk for under-treatment of pain [14]. NH assistant nurses (ANs) reported [15] that it is difficult to determine whether a person’s behaviours are normal personality manifestations or consequences of pain and that the recognition of pain was often a guessing game.

A modest significant increase in adherence to recommended practices over time, i.e. pain assessment and management, was found in a recent mixed-method intervention study that focused on the adoption of evidence-based pain management in NHs [16]. According to Rycroft-Malone et al. [17] facilitators play a key role helping others understand what changes need to be made. Gagnon et al. [18] evaluated a pain training video program in NHs that included both pain assessment and management, which overall revealed favourable results. Reported barriers for successful implementation were for example resistance to change and time restraints. Another cited problem is the lack of nursing documentation in the NHs, which makes it difficult to assess changes in pain symptoms [19]. Continuing pain education has been found to increase documentation [20], which in turn has been associated with reduced pain among residents [21].

**Fig. 1:**
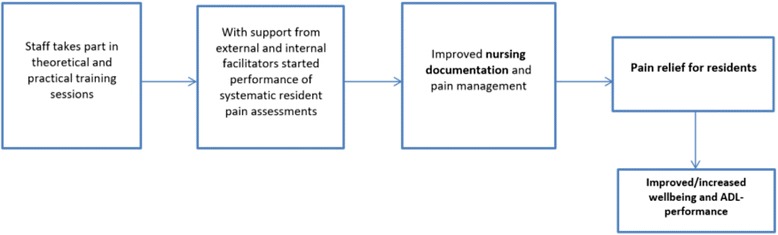
Program logic assumption with measured outcomes showed in bold

Herman et al. [22] proclaimed that prospective intervention strategies are required to improve NH pain management. They stressed that NH pain management requires multifaceted investigative approaches. A recent review [23] regarding interventions to change staff care practices associated with improvement of NH residents’ outcomes, showed that no single program component or an increased number of them improved the likelihood of positive outcomes. Positive resident outcomes tended to change staff behaviour. Changes of staff behaviour did not automatically improve the residents’ outcomes, but tailored specific care tasks provide a greater chance of yielding positive outcomes. The authors empathize that possible barriers and the feasibility of every programme component needs to be considered in order to achieve intended outcomes. Systematic approaches [23] are needed where the components of the process are followed and addressed in order to illuminate the mechanisms of successful interventions. The present study focuses on the outcomes of NH residents regarding wellbeing, pain changes over time, and ADL-dependency as well as those of the nurses’ perceptions of the systematic use of pain assessments scales. Our assumptions were that with systematic pain assessment there will be an increase in the staffs’ awareness of pain among residents, better documentation regarding pain will occur, improved pain management will develop, and a decreased level of pain will result which, in turn, may improve the NH residents’ wellbeing and ADLs, see Fig. 1.

To our knowledge, few previous intervention studies with a pre- and post- design using mixed-methods have described the simultaneous effects the implementation of a 7-month pain management intervention has on NH residents and the staffs’ practices and perspectives. The overall aim of the present study was to investigate the effects of a pain management intervention on NH residents and to describe the staffs’ experiences of the intervention.

The study hypothesis was: Residents living in nursing homes where the intervention is implemented will have lower levels of pain (proxy-measured), and their wellbeing and ADL will be improved over time compared to a comparison group.

Complementary research questions were: 1) How many residents in the intervention group have clinically significant increased pain scores, unchanged, or clinically significant decreased pain scores? 2) How is pain/pain management addressed in the nursing documentation among residents that over time show clinically significant increased pain scores? 3) How is drug use addressed over time? 4) How do the staff members experience the pain intervention?

## Methods

The present study is part of a larger Swedish pain management project performed in 2012 that was mandated to the management of elder care services within a municipality to address and improve pain management in NHs. The pain intervention project was planned with the intention to support development of the pain assessment process and to promote systematic pain management practices and procedures.

### Design

**Fig. 2:**
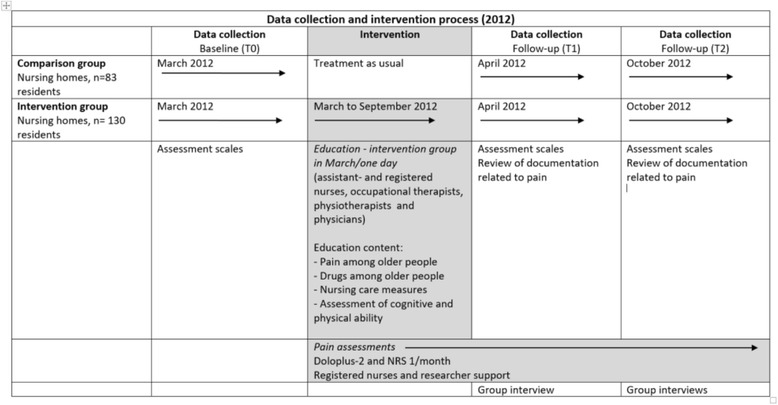
Study design

A cluster-randomized trial was performed and a mixed-methods approach was used with the intention to describe and understand quantitative results more in breadth by exploring qualitative views [24]. The intervention group received theoretical and practical training regarding chronic pain among NH residents; over time assessment scales were used, nursing records were reviewed and nurse interviews were conducted to follow the intervention (Fig. 2).

### Setting

The study was conducted in a municipality with 25,000 inhabitants in central Sweden. There were 13 NHs with a total of about 500 residents in the municipality, all of which were governed by one elder care services management office. In Sweden municipalities are responsible for providing care to older persons based on their health and nursing care needs. Persons residing in NHs have extensive care needs related to health issues and functional decline, for which healthcare personnel are made available 24 h a day. In the municipality from this study the staff to resident ratio was similar to the national average, with staffing during the weekdays and week-ends being 0.33 AN/resident and 0.24 AN/resident, respectively. Corresponding figures for RNs during weekdays and week-ends were 0.04 RN/resident and 0.01 RN/resident, respectively with the RN having a consultant advisory role. Most of the RNs had a Bachelor of Science degree in nursing and the ANs an upper secondary education. Occupational therapists, physiotherapists and physicians were available for all NHs in the municipality once a week, as well as being on call if additional support was needed.

### Sample

**Fig. 3:**
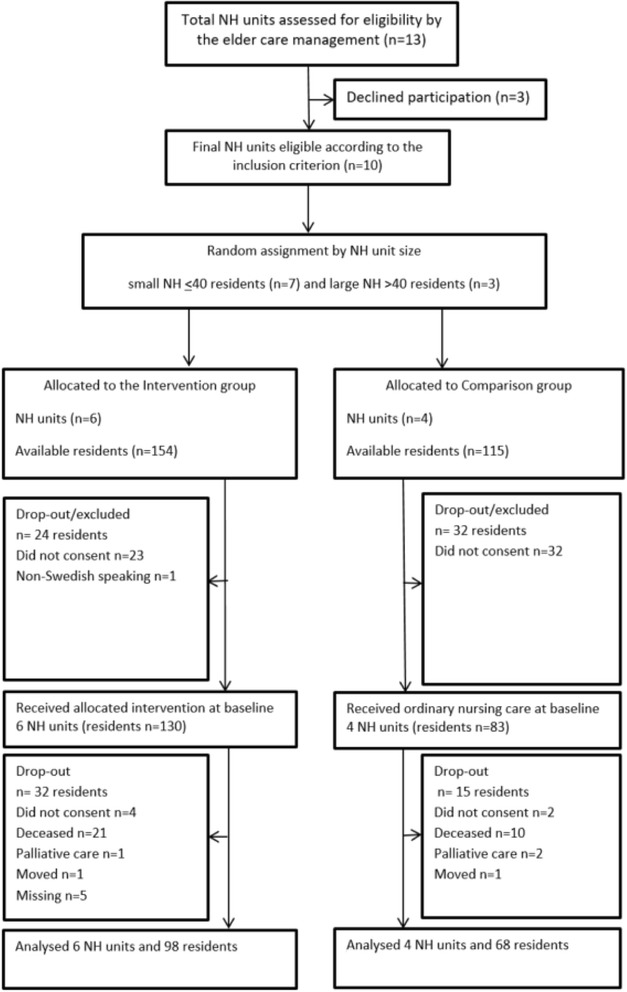
Flow chart of randomizing units and participants

All 13 nursing homes in the municipality were invited to participate; three declined due to other obligations. All residents who resided in the NH on a permanent basis for one or more months except those there on a short term basis or those with palliative care status were approached. Included NH units, residents and drop outs before and during the intervention period are described in Fig. 3.

In the qualitative part, two samples of staff were included. In Time 1 (T1), a group interview with a convenience sample of 45 RNs/ANs took part. These nurses were those who worked on the day that the interview was performed. They voluntarily took part and represented all of the NHs in the intervention group. In Time 2 (T2), the group interviews consisted of a purposive sample of five RNs and six ANs, who had participated during the entire intervention period and had performed several pain assessments. These RNs and ANs worked in six of the NHs.

### Procedure

The ten NHs were divided into two sets according to their size. Small NHs, housing ≤40 residents (*n* = 7) and large NHs housing >40 residents (*n* = 3) formed one set each respectively. Thereafter, the NHs in each group were randomly assigned to either the intervention or comparison group. The randomizing process took into consideration the possibility of drop-out threats and related aspects to maintain statistical power in the intervention group.

### Data collection- residents

Resident data were collected at baseline (T0) and at the two follow-ups (T1 and T2) after the intervention period (Fig. 2). Primary outcome measures were wellbeing and RNs and ANs estimation of the patient’s pain. Secondary outcome measures were ADL-dependency and pain by the nursing documentation reviewed. Background data collected was age, gender, and cognitive status. The scales regarding wellbeing, ADL-dependency and cognitive function were administrated by two of the researchers. The pain assessments were administrated by the RNs and ANs if the residents were not able to express themselves. The residents received oral and written information and signed an informed consent form. For those residents who were unable to sign an informed consent form, a consent form was signed by a relative.

### Instruments

#### Primary outcome measures

##### Wellbeing

The QUALID-scale was used [25], and it measures quality of life among persons having severe dementia disease. It is an 11 item scale that measures the prevalence of a person’s observable behaviours and mood the previous week e.g., smiles, seems to be sad, cries, show signs of being dissatisfied, unhappy or discontented (complains, moans, shouts), is irritated or aggressive (gets angry, swears, violent toward others), likes to eat, and seems calm/harmonic. Five response alternatives (1–5 points) are available for each question with a total sum between 11 and 55 points (11 = highest; 55 = lowest level of quality of life). The QUALID-scale has shown satisfactory psychometric properties Cronbach’s *α* value 0.74 and correlation with other instruments 0.64 (Global Well-Being scale), 0.69 (General Behavior Assessment Scale) and 0.74 (Patient Mood Assessment Scale) [25].

The WHO-5, is a self-report wellbeing index [26] that includes five items: feels cheerful and in good spirits, feels calm and relaxed, has felt active and vigorous, awoke feeling fresh and rested, and considers their daily life has been filled with interesting things. The feelings, which are to reflect the previous 2 weeks are scored on a 6-point scale that ranges between 0 and 5 (0 = not present; 5 = constantly present). The scores are transformed to a score ranging 0–100 (0 = worst; 100 = best thinkable); at 50 points poor emotional wellbeing is suggested and at 28 depressions are indicated. The scale has an adequate validity as a screening tool [27]. For the Who-5 wellbeing index a content validity ratio (CVR) of 0.80 is reported [28].

##### Pain

Proxy-NRS was collected by the staff in both groups as a pain outcome measure. NRS a unidimensional numeric 11-point scale [9] measuring pain intensity within the last 24 h, and is numbered from 0 to 10 (0 = no pain; 10 = worst imaginable pain). The NRS-scale construct validity correlates to the visual analog scale, ranging from 0.86 to 0.95 [29].

The pain assessment instruments that were used only *in the intervention group* were the NRS-scale for self-assessment, and for persons with reduced cognitive ability the Doloplus-2 scale. The Dolplus-2 scale [10] is an observational scale consisting of ten items grouped under three subgroups: 1) somatic (somatic complaints, protective body postures adopted at rest, protection of sore areas, expression, and sleep patterns), 2) psychomotor (washing and/or dressing, and mobility) and 3) psychosocial (communication, social life, and problems with behaviour). Each item is scored in progressive levels of pain-related behaviour from 0 to 3 (0 = normal behaviour; 3 = highest level of pain-related behaviour). The scale ranges from 0 to 30 points (higher scores = more pain-related behaviours), and the cut-off score ≥ 5 indicates pain. The internal consistency [30] showed Cronbach’s *α* value for the total scale of 0.71, the psychomotor reactions scale 0.80, the psychosocial reactions scale 0.78 and for the somatic reactions scale 0.60. The pain examinations were administered by RNs and ANs who knew the resident well.

### Secondary outcomes measures

#### ADL-dependency

The Katz-ADL hierarchical scale, an often used measure in older adults [31], assesses the individual’s functional dependency regarding six basic activities of daily living: bathing, dressing, going to the toilet, transferring, continence and feeding. Definitions used in the scale are: independent = no assistance, partially dependent = needs assistance in one to two activities, dependent = needs assistance in three or more activities. Scores range from 0 to 6 with higher scores indicating more dependency [32].

ADCS-ADL-sev. scale [33] is a 19 item Alzheimer disease specific assessment scale for those with moderate to severe dementia (MMSE 0–15 at baseline) that measures a person’s ability to function and perform ADLs combined with their physical and cognitive abilities. Results are expressed in scores ranging 0–54 with higher scores indicating a higher level of functioning. Interviews are used to assess a person’s ability to perform activities of daily living (personal care, communication and interaction with others, maintaining a household, conducting hobbies and interests, and making judgements and decisions). Concurrent validity of the ADL-sev. and the global function were 0.63 (vs. Clinical Dementia Rating scale, CDR), 0.77 (vs. Clinical Dementia Rating sum of Boxes Scores, CDR-SB) and 0.71 (vs. Geriatric Depression Scale, GDS) [33].

Mini-Mental-State Examination (MMSE) was used as a background variable reflecting the residents ‘cognitive capacity characteristics with a range between 0 and 30 where a score of 30 indicates no impairment in cognition [34]. Two of the researchers who are familiar with the MMSE administered the MMSE examinations.

#### Nursing documentation

Nursing documentation related to pain and descriptions of it such as possible symptoms of pain, drug use and nursing care measures was reviewed from the intervention group. Reviews of records were performed if a resident was assessed as having increased risk for pain or had pain at T1 and/or T2 and the data was collected 3 weeks retrospectively. A specialist nurse in elder care with interest and experience in chronic pain together with two of the researchers performed the nursing documentation reviews.

#### Data collection - staff

The *T1 group interview* was performed after a 2-week practical training period and after the completion of four assessments (Fig. 2). RNs and ANs were interviewed together and the questions addressed were: their thoughts on using the Doloplus-2 scale, the scale’s items, reporting pain or the risk for pain in others, and pain documentation. The interview was conducted at the municipality’s administrative centre and took place on two occasions. The interview lasted between 20 and 35 min, was tape-recorded and transcribed verbatim. Oral and written information had been provided to the RNs and ANs prior to the proposed group interview T1, and for those who participated, it was assumed that that they had given informed consent.

The *T2 group interviews* were performed after the intervention period (Fig. 2). Interviews with the RNs and ANs were conducted separately [35], and focused on their experiences of their participation in the pain management intervention. Questions addressed were: how do you reflect on your ability to identify pain or risk for pain, what are your experiences with using the pain assessment scales, and what are your thoughts on pain documentation. The interviews lasted from 75 to 100 min, were tape-recorded and transcribed verbatim. The managers provided oral and written information about the T2 group interviews and at the time of the interview an informed consent form was signed.

At both T1 and T2 structured interviews were performed using a guide as well as clarifying probes. Conducting the interviews was one researcher that assumed the role of moderator and one that was an assistant [35]. One of the experts who performed the theoretical intervention participated at the group interviews (T1) to elaborate on the questions.

### Intervention

The intervention was performed over a 7-month period from March 2012 to September 2012. Staff from the intervention group were invited to attend four, 4-h theoretical and practical training sessions*. The theoretical training* was a presentation of evidence-based knowledge regarding the pain problems related to older people, types of pain, medical diagnoses, pain assessments for persons with and without cognitive impairment, drug treatments, nursing measures that can alleviate pain and discussions on how to improve pain management. The theoretical training was provided by one RN/associate professor and one Physician/professor, who were experts in the fields of pain and dementia disease among older people. The pain assessment scales used were the Doloplus-2 scale [10], which was new for the staff and the Numeric Rating Scale (NRS-scale) [9] that had been used at the NH, but not routinely. The theoretical training contained a brief introduction of the Doloplus-2 scale assessment, which was presented and discussed in smaller groups together with four external facilitators [10]. These external facilitators were appointed by elder care services management to support the NH staff. They had special interest in pain management, were specialists with postgraduate degrees in geriatric nursing and worked as RNs within the municipality, but not at the NH they were going to support.

After the theoretical introduction a *practical training period* started within 2-weeks. The assigned staff performed at least four Doloplus-2 pain assessments each during the training period and supported residents to rate pain on the NRS-scale when possible. Before the start of the practical training period, the four external facilitators visited all of the NHs to discuss possible uncertainties regarding the scales or the assessments. At each NH internal facilitators were also appointed as daily supporters of the intervention process, they were assigned by their managers after voicing an interest and desire to share in the responsibility of the intervention’s implementation. There were 1–2 RNs or 2–6 ANs per NH, depending on the size of the NH. The use of external and internal facilitators was inspired by Rycroft-Malone et al. [17] in that facilitators could enable individuals and teams to reflect on their attitudes, behaviours, ways of working and if any changes were needed.

During the entire intervention period, the external facilitators visited the NHs once or twice a week at the beginning and then once a week for the remainder of the intervention. Researchers made physical or telephone follow-ups twice a week during the first months, and later twice a month to the NHs. The researchers met with the external facilitators regularly during the intervention period. In total, there were about 380 staff members consisting of RNs, ANs, occupational therapists, physiotherapists and physicians that took part in the training. After the intervention, the comparison group also received theoretical and practical training.

### Data analysis

#### Resident data

Since some units had very few residents participating with some of the instrument (between 1 to 42 residents/unit), we first analysed the data on a residential level and thereafter we used generalized estimating equation (GEE) models controlling for clustering effects to see weather this changed our results or not. On the residential level, Wilcoxon Signed Rank-Test was used for within-group comparisons over time and Mann–Whitney *U*-test for between-group comparisons at baseline and to compare the differences in change score over time between the intervention and comparison group. Student’s independent *t*-test was used for continuous data and Chi2–test was used to detect differences in the frequency data at baseline. In the GEE models, independent variables were main effect for time and main effect for group and interaction effect, i.e. time*group. Where there were statistically significant differences between the two groups at baseline we controlled for baseline values in the GEE models. GEE analyses take advantage of all values in its estimates and thereby also takes into account values for residents that later drop-out [36]. Statistical significance levels were set at *p* ≤ 0.05 (two-tailed). A changed pain score of 30% (clinically relevant) [9] was used when analysing the pain assessments, which then was measured as changes in scores in the intervention group. Statistical analyses were performed using SPSS 22.0 (SPSS Inc., Chicago, IL).

### Review of nursing documentation

Records were reviewed among residents that over time (T1 and T2) showed changed pain assessment scores of ≥ 30% (clinically relevant increase) [9] according to the Doloplus-2 scale and the Numeric Rating Scale, i.e. indicating risk for pain or reported pain. The focus for this review was the nursing documentation related to pain or pain management.

### Staff interview data

The group interviews (T1 and T2) were analysed using qualitative content analysis [35]. T1 and T2 were analysed separately. An inductive approach was used when analysing the data. Entire texts from the respective interview occasions were taken into consideration and read through several times. The meaning units were then identified, condensed, coded and sorted based on similarities and differences into subcategories and categories. Similarities and differences were reflected on during the analysis process and results were discussed until a consensus was reached [35, 37].

## Results

In total, six units with 98 of 130 residents, and four units with 68 of 83 residents from the intervention and comparison group respectively, participated at baseline and follow-up (T2). Included NH units, residents and drop outs before and during the intervention period are describe in Fig. 3. The mostly female sample 71.5% (intervention group) and 63.9% (control group) included residents of high age mean 85.5 SD 6.7 (intervention group) and mean 85.1 SD 7.2 (control group) with considerable cognitive impairment md 11.5; Q1-Q3, 0.75–19.25 (intervention group) and md 16; Q1-Q3, 0–21.0. Characteristics of the residents in the intervention and comparison groups at baseline are seen in Table 1. There were significant differences at baseline regarding wellbeing/WHO-5 index (*p* = 0.003), ADL-dependency/Katz-ADL (*p* = 0.013) and ADCS-ADL (*p* = 0.032) with higher wellbeing, lower dependency measured with Katz-ADL and higher dependency measured with ADCS-ADL (measuring both physical and cognitive ability) in the comparison group compared to the intervention group. For the other variables and background data there were no statistically significant differences at baseline (Table 1). Drop-out analyses showed significant differences between drop-outs and participants for WHO-5 wellbeing index (*p* = 0.019), Katz-ADL (*p* = 0.027), ADCS-ADL (*p* = 0.029).

**Table 1:** Characteristics of participants in the intervention and comparison groups (*N* = 213) at baseline (T0)

Characteristics	Intervention group (*n* = 130)	Comparison group (*n* = 83)	*p*-value
Age in years, n 130/83, mean (SD)	85.5 (6.7)	85.1 (7.2)	0.694^g^
Female gender, n (%)	93 (71.5)	53 (63.9)	0.239^h^
MMSE^a^ n 130/83, md (Q1-Q3)	11.5 (0.75–19.25)	16 (0–21.0)	0.756^i^
QUALID^b^ n 75/44, md (Q1-Q3)	21.0 (18.0–27.0)	24.5 (18.0–30.75)	0.240^i^
WHO-5^c^ wellbeing index n 56/40 md (Q1-Q3)	60.0 (44.0–80.0)	78.0 (57.0–88.0)	0.003^i^
Proxy NRS^d^ n 130/82, md (Q1-Q3)	3.0 (1.0–6.0)	3.0 (1.75–6.0)	0.641^i^
Katz-ADL^e^ n 129/83, md (Q1-Q3)	5.0 (2.5–6.0)	3.0 (1.0–6.0)	0.013^i^
ADCS-ADL^f^ sev n 79/40, md (Q1-Q3)	10.0 (5.0–21.0)	5.5 (3.0–12.0)	0.032^i^

^a^Mini-Mental-State Examination (MMSE). ^b^Qualid scale: Quality of Life in Dementia Scale, an observational scale, range with minimum score 11 indicating high QOL and maximum score 55 indicating poor QOL. ^c^WHO-5 index: WHO-5 well-being index: self-report, score from 0 (worst thinkable well-being) to 100 (best thinkable well-being). ^d^Numeric Rating Scale: self-report, from 0 (no pain) through 10 (worst imaginable pain). ^e^Katz index of ADLs: score from 0 to 6, higher score indicating more dependency.^f^ADCS-ADL: Alzheimer’s Disease Cooperative Study Activities of Daily Living Scale, scale from 0 to 54 points with lower scores indicating more dependency

*Md* median, *Q* quartil, ^g^ Student’s independent *t*-test, ^h^ = Chi2-test, ^i^ = Mann–Whitney *U*-test

In the T1 group interviews 45 RNs/ANs (one male) participated. In this sample no background characterises were collected. In the T2 group interviews five RNs and six ANs participated. The RNs, all female 35–60 years of age; mean 46.2 SD 9.6, had worked 7–40 years; mean 16.0 SD 13.2 as RNs and some had taken additional university nursing courses in e.g., palliative and dementia care. The ANs were all female and aged between 34 and 57 years; mean 50.3 SD 9.1 had worked 14–38 years; mean 20.2 SD 8.9 in patient care and some reported university courses in e.g., palliative and dementia care, rehabilitation, and ethics.

### Residents

#### Changes over time within and between the intervention and the comparison groups

##### Primary outcome measures

For the variables: QUALID, WHO-5 wellbeing index and proxy-NRS, no significant differences in change scores over time between the groups or within each group were found (Table 2).

**Table 2:** Changes over time, baseline (T0) and follow-up 2 (T2), in the intervention group (*n* = 98) and comparison group (*n* = 68) concerning wellbeing, proxy-pain and functional dependency

	Intervention group, median (Q1, Q3)		Within group^f^	Comparison group, median (Q1, Q3)		Within group^f^	Changes over time between the groups^g^
Measurements, n intervention/comparison	T0	T2	*p*-value	T0	T2	*p*-value	*p*-value
QUALID^a^ 53/32	21 (17–27)	22 (17–28)	.409	23.5 (17.25–29.75)	22,5 (17.0–27.75)	.965	.733
WHO-5 wellbeing index^b^ 24/31	64 (50–80)	68 (46–83)	.665	76 (56–88)	76 (64–88)	.965	.683
Proxy-NRS^c^ 97/67	3 (1.0–5.25)	2 (0–5.5)	.396	3 (1–6)	4 (1–6)	.711	.309
Katz-ADL^d^ 98/66	4 (2–6)	5 (2–6)	.638	3 (1–5)	5 (2–6)	**<.001**	**.001**
ADCS-ADL sev.^e^ 60/33	10.5 (5–21.75)	9 (4.25–15.75)	**.011**	6 (4–20.5)	7 (5–13)	.498	.297

*Q* quartil, ^a^Qualid scale: Quality of Life in Dementia Scale, an observational scale, range with minimum score 11 indicating high QOL and maximum score is 55 indicating poor QOL. ^b^WHO-5 index: WHO-5 well-being index: self-report, score from 0 (worst thinkable well-being) to 100 (best thinkable well-being). ^c^Numeric Rating Scale: self-report, from 0 (no pain) through 10 (worst imaginable pain). ^d^Katz index of ADLs: score from 0 to 6, higher score indicating more dependency.^e^ADCS-ADL: Alzheimer’s Disease Cooperative Study Activities of Daily Living Scale, scale from 0 to 54 points with lower scores indicating more dependency. ^f^ Wilcoxon Signed Rank-Test ^g^Mann-Whitney *U*-test

##### Secondary outcome measures

For ADL-dependency measured with Katz-ADL the results showed a statistically significant difference in change over time between the two groups (*p* = 0.001), with a significant increase in dependency in the comparison group over time (*p* < 0.001), Table 2. However, when both physical and cognitive function was measured using ADSC-ADL-sev. as a combined scale for a subgroup of patients with MMSE 0–15 at baseline, there was a significant decline in physical and cognitive function over time in the intervention group (*p* = 0.011; *n* = 66). Although, there was a non-significant interaction effect, i.e. changes over time between the two groups were non-significant. For this subgroup, persons with MMSE 0–15 at baseline in the intervention group, there was also a tendency for a deterioration in cognitive function measured with MMSE (*p* = 0.070).

The results regarding baseline to follow-up 1 showed similar results as baseline to follow-up 2, and therefore only scores for the last follow-up are presented in Table 2. For baseline to follow-up 1 there were also statistically significant differences in change scores between the two groups in Katz-ADL (*p* = 0.05), with a significant increase in dependency in the comparison over time (T0 median [md] 3.0, quartile [Q] Q1-Q3 1.0–5.25; T1 md 4.0, Q1-Q3 2.0–6.0; *p* = 0.001). For the other variables there were non-significant results over time within groups as well as change over time between the intervention group and comparison group when analysing baseline to follow-up 1.

Results using GEE-models controlling for potential clustering effects using GEE models the results confirmed earlier analyses. The results showed significant interaction effects, i.e. changes over time differed between the two groups, for Katz-ADL (*p* ≤ 0.001); and non-significant results for QUALID (*p* = 0.853), WHO-5 wellbeing index (*p* = 0.633), PROXY-NRS (*p* = 0.314) and ADCS-ADL (*p* = 0.643), Table 3. Regarding Katz-ADL there was significant increased dependency at T1 (*p* = 0.001) and T2 (*p* ≤ 0.001) compared to T0 for the comparison group while for the intervention group there were no significant changes in dependency over time in the residents’ Katz-ADL (Table 3).

**Table 3:** Parameter estimates and 95% CIs from GEE analyses for the scales, QUALID, WHO, PROXY-NRS, Katz-ADL and ADCS-ADL sev. scale

	QUALID		WHO^a^		PROXY-NRS		Katz-ADL^a^		ADCS-ADL sev	
	B	*P*-value	B	*P*-value	B	*P*-value	B	*P*-value	B	*P*-value
GEE Model										
Interaction time*group	0.34 (−3.30;3.99)	0.853	−2.30 (−11.74;7.14)	0.633	−0.45 (−1.34;0.430)	0.314	−0.84 (−1.28;−0.41)	**≤0.001**	−0.55 (−2.88;1.78)	0.643
Mean differences										
Intervention group T0-T1	0.69 (−1.12;2.49)	0.456	−7.52 (−15.7;0.65)	0.071	−0.23 (−0.81;0.34)	0.427	0.06 (−0.17;0.29)	0.617	0.07 (−1.50;1.64)	0.931
Intervention group T0-T2	0.74 (−1.41;2.89)	0.502	−1.00 (−8.34;6.35)	0.790	−0.37 (−0.99;0.25)	0.242	0.02 (−0.27;0.30)	0.908	−1.41 (−2.80;−0.02)	0.047
Comparison group T0-T1	0.17 (−2.29;2.63)	0.895	2.98 (−3.61;9.57)	0.376	−0.39 (−0.97;0.19)	0.186	0.46 (0.19;0.72)	**0.001**	−0.60 (−2.16;0.96)	0.450
Comparison group T0-T2	0.39 (−2.55;3.34)	0.794	1.30 (−4.89;7.50)	0.680	−0.09 (−0.72;0.55)	0.793	0.86 (0.53;1.18)	**≤0.001**	−0.86 (−2.73;1.01)	0.369
Intervention group T0 – Comparison group T0	−1.40 (−4.39;1.59)	0.360	−3.02 (−5.21;−0.83)	**0.007**	−0.092 (−0.84;0.65)	0.808	0.13 (0.03;0.23)	**0.011**	3.07 (−0.67;6.81)	0.107

*CI*confidence interval, bold print indicates statistically significant values, ^a^for WHO and Katz-ADL we controlled for baseline (T0) data in the GEE models as there were significant differences between the intervention and comparison groups at baseline. QUALID scale: Quality of Life in Dementia Scale, an observational scale, range with minimum score 11 indicating high QOL and maximum score is 55 indicating poor QOL. WHO-5 index: WHO-5 well-being index: self-report, score from 0 (worst thinkable well-being) to 100 (best thinkable well-being). Numeric Rating Scale: self-report, from 0 (no pain) through 10 (worst imaginable pain). Katz index of ADLs: score from 0 to 6, higher score indicating more dependency. ADCS-ADL: Alzheimer’s Disease Cooperative Study Activities of Daily Living Scale, scale from 0 to 54 points with lower scores indicating more dependency

### Change of pain assessment scores over time in the intervention group at an individual level and in the nursing documentation

At T1, 62% of the residents (*n* = 52) in the intervention group were at risk for pain measured by the Doloplus-2 scale (cut off score, ≥ 5 points) and at T2 corresponding figures were 69% (*n* = 36). Regarding changes of pain assessment scores of ≥ 30% according to the Doloplus-2 scale (*n* = 44, missing *n* = 48) between T1 and T2, 17 residents showed increased pain scores ≥ 30%, indicating they were at risk for pain or in pain. Of these 17 residents, eight of them were identified with pain at T1. Fourteen of the residents did not have ≥ 30% change in either direction, while 13 residents showed decreased scores during the period. Pain reported by measuring with the NRS (*n* = 7, missing *n* = 85) showed that two residents had increased pain intensity scores of ≥ 30% over time and one of these had also reported pain at T1, two showed decreased scores and three residents reported neither an increase nor decrease of pain scores ≥ 30% (unchanged scores).

Nursing documentation was reviewed among the 19 residents identified with increased pain scores ≥ 30% T1 to T2. Nursing documentation related to pain reactions was found among five of the 19 residents. The nursing documentation described briefly the resident’s reactions and/or analgesic or sedative drugs provided, while other pain management descriptions were sparse.

### Change of drug use over time in the intervention and comparison groups at a group level

No significant changes in the use of analgetics was found in any of the groups. Similar pattern was seen in other drugs which regimen can be potentially harmful if not used with caution (e.g. anticolinergic drugs) (Table 4).

**Table 4:** Change of drug use over time in the intervention and comparison groups at a group level

	Time T0			Time T2		
Measurements, n intervention/comparison	Intervention group, n (%) user	Comparison group, n (%) user	*p*-value	Intervention group, n (%) user	Comparison group, n (%) user	*p*-value
Analgetics	99 (79.8)	54 (72.0)	.204	43 (89.6)	30 (78.9)	.171
Long acting Benzodiazepines	13 (10.5)	10 (13.1)	.417	4 (8.3)	5 (13.2)	.468
Anticholinergics	16 (12.9)	13 (17.3)	.527	4 (8.4)	4 (10.5)	.469

Md Q1_Q3

### Staff

The nurses’ experiences and perceptions over time from participation in the pain intervention: Results from group interviews (T1 and T2) are presented in nine subcategories and three categories. Quotations from the interviews are provided (Table 5).

**Table 5:** Results from the group interviews (T1 and T2) presented in nine subcategories and three categories

Interviews	Quotations from the interviews	Subcategories	Categories
Group interview (T1)	‘X: The facial expressions and the sleep X: The facial expression is the best, then you can see that wrinkle there and the whole thing, the first physical symptoms’.	The scale works well but is not always necessary	A new way of working to identify pain
	‘For temporary fill in personnel it is another thing entirely, the temporary staff barely know them [the residents]’.		
	‘X Maybe get the staff’s interest going, so that everyone strives towards the same goal. X: What can it be when she does like that or is looking like that? When they don’t hear, or they don’t see, they can’t make themselves understood even if it’s not dementia, just as difficult’.	Helps to put a focus on pain	
	‘X: Says that it’s not good today. X: It seems that he may have pain, but there is nothing you can put your finger on’.	Pain assessments can help bring about an improvement in documentation and evaluation	
	‘Can’t make an evaluation after such a short time. Assessing pain can still feel like a problem because doing that is still so new to us, a person needs to train a bit more’.	A longer trial period is needed to evaluate the usefulness of the scale	
Group interviews (T2)	‘X: What do you mean, a little headache, one’s own personal views are incredibly important. X: If one accesses pain for every person, so to say, then you’ll get quite a few different responses. X: Some of the older ones have a high pain threshold, they don’t complain much’.	Be observant of pain indicators and your personal views regarding pain	A systematic method of working and better communication facilitates better pain management
	‘X: We have had several doctors…then there was a summer substitute and then we had a new one again. X: Feels very confusing. X: Now, we have a doctor from the local medical care centre here and he is very dedicated and good to talk to’.	Pain assessments facilitate more effective teamwork and drug treatments	
	‘If a person has made an assessment and it says that someone is likely to have pain, then you have evidence with the pain assessment’.		
	‘It is easier to get the staff to understand that you try other things [acetaminophen] first, it can be pain but it can also be something else that is causing the uneasiness, try something like that, to begin with before some sedative’.		
	‘X: Very different, doing this pain assessment. X: It has taken a lot of time but that will probably get better, it should be easy and there are benefits with it, but of course we don’t get more time either, instead we’ll have to make time to do it’.	Usability of the scale and items and work practices	
	We have considered it to be extremely interesting and thought it was great fun’.	Planning phase, lectures and pain assessment use	
	‘There are staff members that have questioned this, I could also say so, otherwise I would be lying but most of them are positive’.		Possibilities and obstacles with the intervention
	‘X: Really good dialogue, she checks how it’s going, knows exactly how it is’. ‘X: Has not shown interest, but I hope that they [managers] will be interested.’	Manager support	

Nurses’ narratives are denoted with (X)

### Group interview (T1)

#### A new way of working to identify pain

##### The scale works well but is not always necessary

The Doloplus-2 scale was perceived to be adequate and able to capture possible pain indicators among residents that were severely impaired cognitively. Expressions and sleep patterns were two scale items emphasized as particularly useful when observing for physical indicators of pain. The scale items were found to be easy to fill out as the nurses knew the residents rather well. It was highlighted that their familiarity with the residents reduced their need of an assessment scale, even among those that were unable to verbalize their pain. They could quickly read a resident’s reactions and know they indicated pain even without the use of scales. Scales were found useful if the staff was unfamiliar with the residents.

##### Helps to put a focus on pain

Performing assessments was initially perceived as overwhelming, as the workload was already considered heavy. Using pain assessments were at the same time described as something positive, since it could help to increase the focus and interest on pain issues.

##### Pain assessments can help bring about an improvement in documentation and evaluation

Pain management measures were only partly utilised. The ANs and the RNs discussed the residents’ pain, but it was not always documented. The use of pain assessment results were expected to be helpful when making evaluations.

##### A longer trial period is needed to evaluate the usefulness of the scale

The assessment test period [T0 to T1] was described as being too short for the nurses to be able to make an evaluation. Additional time to experience using the scale was requested.

### Group interviews (T2)

#### A systematic method of working and better communication facilitates better pain management

##### Be observant of pain indicators and your personal views regarding pain

It was described that it could be difficult put oneself into another person’s situation and not be steered by one’s own views regarding pain. They stressed that they were more attentive to the residents’ possible pain complaints and reflected on their responses.

##### Pain assessments facilitate more effective teamwork and drug treatments

Team member dialogue increased when the pain assessment results were analysed. During periods of physician shortages, providing pain management was not always easy. RNs reported that the results contributed to more concrete discussions with the physicians. The ANs felt that the results provided evidence that made it possible for them to get the RNs to listen to their opinions*.* RNs reported that the ANs asked less often for sedative drugs to be given right away due to a residents’ possible signs of anxiety. The most common first-line approach was to test an analgesic drug. The nurses reported increased abilities in the residents’ ADLs after an improved awareness regarding possible pain and medicinal pain therapies.

##### Usability of the scale and items and work practices

Working with the Doloplus-2 scale was initially considered time consuming, but gradually it became easier to perform the assessment. The acknowledged benefit was the provision of a structure for pain management. Different work practices were reported as problematic. The lack of nursing pain documentation had led to fewer evaluations of the assessment results. The RNs’ consultant advisory role that was spread out serving several NHs at the same time, limited time and availability for each resident and AN, and prevented follow-ups. The RNs not having full access to all NH nursing records at all times was described as a problem related to the structure of the data system and something beyond their control.

#### Possibilities and obstacles with the intervention

##### Planning phase, lectures and pain assessment use

Some RNs perceived the information given prior to the intervention as being too sparse. They wanted more lectures regarding pain and separate training sessions for RNs only. Some of them had not used pain assessment scales earlier and felt insecure when supporting the ANs. The ANs described that the intervention was rather good, but considered some parts of the lecture too elementary since some of them had experience in palliative pain management. A number of nurses did not participate in the lecture due in part to a shortage of personnel. It was felt that this led to less interest among some of the staff to prioritize the pain assessments.

##### Manager support

It was reported that the managers varied in their support during the intervention period. When it was felt to be lacking there was a strong request for improvement.

## Discussion

### Residents

Our results showed, when comparing the intervention and comparison group, there were non-significant results for pain and wellbeing. However, for ADL-dependency (Katz-ADL) there was a significant increased dependency in the comparison group over time, while group level ADL-dependency in the intervention group remained approximately the same. The results indicate that an increased awareness of pain will contribute to improved pain management and decreased deterioration or maintenance of the physical and functional abilities among NH residents. Lapane [6] described that residents with persistent pain spend less time involved in activities, which in turn increases their immobility and can result in increased dependency in ADLs. However, a significant increased dependency over time was measured in the intervention group when both physical and cognitive ability was measured with the ADSC-ADL-sev. scale among the residents with MMSE 0–15 at baseline. This probably reflects the residents’ cognitive deterioration during the intervention period as the ADSC-ADL-sev. scale also contains items regarding cognitive aspects. Some scale items such as turning off lights, obtaining beverages and using the telephone were considered to be unaffected if pain assessments were carried out and measures were provided. No interaction effect was found for ADSC-ADL-sev, i.e. there were no statistically significant differences in change scores between the intervention and comparison group.

Previous NH studies [4, 6, 8] highlighted aspects such as decreased quality of life and wellbeing related to pain. In the present study, no significant changes in wellbeing over time in either of the groups were described. Consideration needs to be given to the fact that most of the NH residents suffer from multiple illnesses and even if the pain management is tailored, a severe cognitive impairment and depression diagnosis significantly impinges on the wellbeing. Another aspect related to the difficulties of detecting significant changes over time in wellbeing and pain can be a result of our group level presentation, in that comprehensive variation at the individual level is not captured, as was also indicated in our analyses of pain. About half of the intervention group residents assessed by Doloplus-2, had increased clinically relevant changes in pain scores, indicating they were at risk for pain or were in pain. The nursing documentation related to pain reactions or measures provided in the present study, was sparse. Insufficient documentation is problematic as it is a central aspect of patient safety, in that important information will not be available for the team members [19].

### Staffs’ experiences of the intervention

In general, the nurses expressed positive attitudes from the outset of the intervention towards performing pain assessments, which was a work approach considered central to improved pain management. However, performing pain assessments was not always perceived to be necessary. The positive attitudes that persisted over time, seemed to be strengthened by the continued assessment experiences and were associated with the nurses’ perceptions of improved pain management. The generally positive descriptions of the work approach, which included the pain assessments and the team member analyses, might mirror the residents’ decreased deterioration or maintenance of physical and functional abilities found in the quantitative results. The nurses stressed that with improved pain management there was also an increase in the physical abilities of the residents.

It is expected that evidence-based pain recommendations will be followed, and that NH nurses perform pain assessments followed by the necessary treatment (s). In the present study, 62% of the residents were assessed to be at risk for pain at T1 and 69% at T2. These results may perhaps mirror an increased awareness and changed understanding of pain issues over time. However, RNs and ANs in NHs report an uncertainty with pain detection among residents with dementia disease [15]. The perceived intervention benefits yielded in the present study can be understood in light of what Sandberg and Targama [38, 39] describe as an understanding of competence in an organization. The members of a collective reflect and act with respect to the situation as a whole, such as with thoughts of one’s own obligations, reflections about right and wrong and if there are other alternatives available. After the intervention the staff showed new ways of thinking, they reported benefits such as a changed structure in pain management practices and extended team member reflections.

Even if the benefits of the work approach were emphasized in the present study, the problem of insufficient nursing pain documentation was raised and confirmed the results found in the review of the nursing documentation. In the present study, it is possible that the use of assessment scales contributed to the misconception that pain problems and measures were documented. However, insufficient nursing documentation in resident records were found. Not only is the lack of documentation in NHs the problem [19], central is also the quality of the information as it is more than a basis for communication and care decisions. The RNs did not always have access to the medical records at the multiple NHs they were responsible for. Pain management routines should be based on information from self-reports or comprehensive pain assessments shared by the team [40, 41]. In the present study, by reflecting on the pain assessment results, the dialogue between ANs and RNs as well as between RNs and physicians was perceived to be improved. A barrier for ANs communication was the RNs’ consultant advisory role. Karlsson et al. [42] also described how the NH RNs consultant role can cause them to feel out of control regarding pain assessment and documentation. In the present study, most of the NH staff members participated in the pain intervention. However, not all staff members could attend the theoretical training, which had negative consequences in that some of staff members were seemingly less interested to prioritize the pain assessments. The necessity for the refreshment training course in pain management can be understood [18] since education or training is often the first necessary step in an intervention [43]. Ista et al. [44] in a review of how to best implement pain assessment strategies in hospitals, reported that in order to increase the inherent motivation among the staff, one needs to involve local experts that provide feed-back at the individual or unit level. Successful implementation increases if the evidence is robust, the context is receptive to change and the process of change is appropriately facilitated [45, 46]. During the implementation process [17] facilitators have a key role to play in helping others understand what needs to be changed. Surprisingly, in the present study no narratives regarding the support provided by the external facilitators during the intervention period were given. This may be related to the fact that the external facilitator nurses were former colleagues and may have therefore been considered to be a part of the work team.

The managers understanding of clinical practice issues are important for the implementation of nursing guidelines [47, 48]. In the present study, the managers for the most part were perceived by the RNs to be supportive. According to Sandberg and Targama [39] the managers often when trying to implement a change process expect the goals to be rapidly met, while the nurses meet the residents face to face and try to fulfil their nursing care obligations on a more personal level. Therefore, it is important that the managers have an ongoing dialogue on the work approach during the implementation process in order to maintain the nurses’ positive attitudes. This is important because it is the nurses that have actual information regarding the pain status of each resident.

### Strengths and weaknesses of the study

The strength of the present study was its design; a cluster-randomized trial and mixed-methods (quantitative and qualitative data collection). The design allows reflections on, and can encompass the understanding of, the quantitative results revealed over time, and all in light of the qualitative views. Prospective intervention studies are required to improve NH pain management [23]. However, studies within the domain of pain and with this kind of mixed-methods approach have not been found. There are weaknesses in the study; the sample was randomized at the NH level, not at the individual level, which might have affected the initial differences between the intervention group (more functional dependency) and the comparison group (better wellbeing). To control for potential clustering effects within units we also performed GEE models that confirmed the results from the residential level. In these tests we also controlled for differences between the two groups at baseline. The sample was small, and the data were presented at a group level, which can contribute to the loss of individual differences over time. However, results from the intervention group were presented at the individual level, which was necessary due to the nursing documentation review performed.

There might have been threats such as diffusion of the intervention to the comparison group. Further research would be needed to confirm if diffusion did or did not actually occur. Another strength was the group interviews that were conducted with the staff, since they are the ones that meet residents in their daily work situation. Interviewing on two occasions; pre- and post -intervention (T1 and T2) was found valuable in that intervention processes could be followed. The interview (T1) created possibilities to make adaptations if needed early in the intervention phase. No changes were needed and the intervention was therefore performed as planned. Longitudinal qualitative research is advised for following processes [49]. It is important to keep in mind that among the nurses interviewed at both occasions (T1 and T2), were the assigned nurses who voiced an interest in taking part in the intervention at the outset, as well as their colleagues. Furthermore, the registered nurses and the assistant nurses were interviewed in two separate groups, which probably led to open narratives. One weakness was that no physician participated in the interviews. During qualitative content analyses author co-assessments are important [34]. In the present study author co-assessments were made in order to ensure consistency throughout the analysis phase.

## Conclusion

The implementation of a systematic work approach to pain issues in nursing homes indicates that an increased awareness, collaboration across and shared understanding among the team members of the pain assessment results can improve pain management and lead to decreased physical deterioration or the maintenance of physical and functional abilities among NH residents. However, pain (proxy-measured) and wellbeing level did not reveal any interaction effects between the groups over time.

## Abbreviations

ADCS-ADL-sev scale: Alzheimer’s disease cooperative study activities of daily living scale
ADL: Activities of daily living
AN: Assistant nurses
Chi2: Chi-square test
GEE: Generalized estimating equation
Katz-ADL: Katz-ADL hierarchical scale
MMSE: Mini-mental-state examination
NHs: Nursing homes
NRS-scale and Proxy-NRS: Numeric rating scale
QUALID-scale: Quality of life in dementia scale
RNs: Registered nurses
T1: Follow-up 1
T2: Follow-up 2
WHO-5 index: WHO-5 well-being index
